# Force majeure impact on citizen science: Perspective from an EU funded project

**DOI:** 10.12688/openreseurope.19184.1

**Published:** 2025-03-03

**Authors:** Huma Shah, M. Pocs, J. Vallverdu, M. Hinsenkamp, Tiberius Ignat, Maayan Zhitomirsky-geffet

**Affiliations:** 1 Coventry University, Coventry, England, UK; 2 Stelar Security Technology Law Research UG, STELAR, FANNY-LEWALD-RING, HAMBURG, 21035, Germany; 3 Universitat Autonoma de Barcelona, Barcelona, Catalonia, 08193, Spain; 4 NOK A TUDOMANYBAN EGYESULET, BUDAPEST, 1121, Hungary; 5 Immer Besser GMBH, MUNCHEN, 81245, Germany; 6 Bar Ilan University, Ramat Gan, 52900, Israel

**Keywords:** apps, citizen science; COVID-19; cyberattacks, digital services act (DSA), diversity, force majeure, inclusivity, informal education, informed consent, GDPR, legitimate interest, mental health, pandemic effects, transparency, websites

## Abstract

**Background:**

Management of citizen science engagement during a force majeure needs very careful consideration in order not to lose precious time. This paper serves as a guide for pro-action in the event of another virus-enforced stay-at-home, movement-control order and presents necessary changes undertaken by an international collaboration during a once-in-a-hundred years’ pandemic that reduced face-to-face public interactions. Overcoming challenges imposed under force majeure conditions, a citizen science project funded under the EU Horizon2020 research and innovation scheme SwafS15-2019, rethought experiential learning through volunteering screen time.

**Methods:**

Mitigation measures, to limit the handicap of moving to online working reducing potential for learning through synchronous live engagement, included creation of an interactive multilingual informal five-step learning resource (CSI-COP MOOC: ‘Your Right to Privacy Online’), adapting Greenpeace’s ‘Big Plastic Count’ to an online ‘Big Cookie Count’ event, as well as webinars organised by project partners in their local language, Newsletters, provided opportunities for the general public to gain vital knowledge about protecting personal data and preserving online privacy.

**Results:**

Over one-hundred and ninety members of the public who completed CSI-COP’s MOOC with a certificate, participated in one-to-one online ‘walk through’ training sessions in local languages joined the project, through GDPR-compliant written informed consent, as citizen scientists. The acquisition of practical skills by these individuals enabled investigations of websites they visited and apps they used to record third-party cookies or third-party requests for personal data.

**Conclusions:**

The effective reorganisation of CSI-COP activities to online, then hybrid once COVID-19 restrictions were lifted, ensured the expected deliverables were produced. Citizen scientists’ contributions realised a searchable Repository of investigated websites and apps, a Taxonomy of tracking cookies, two policy briefs on improving monitoring of GDPR compliance, and Guidelines to address the ‘legitimate interest’ principle used by third parties to gather personal data online.

## Plain Language Summary

Citizen science is a democratic pursuit affording opportunities for the general public to contribute to advancing scientific knowledge and policy-making through collaboration with scientists. The once-in-a-hundred-years COVID-19 pandemic set challenges for an EU funded international project exploring GDPR compliance through the extent of online tracking in websites and apps. This article is relevant, not only because of the likelihood of another pandemic in less than a hundred years, but also the issues of data protection and privacy are real with compliance strengthened in the EU's Digital Services Act

## Introduction

This paper presents a case study of challenges and solutions to conducting citizen science research and innovation activities throughout a 21 ^st^ century force majeure. During the execution of a project funded under the EU Horizon2020 ‘Science with and for Society’ (SwafS) theme (grant agreement GA 873169) ( EU Cordis (2019), ‘Citizen Scientists Investigating Cookies and Apps GDPR Compliance’ ( CSI-COP), restrictions on mobility and public gatherings in project partner countries seriously affected the organisation and hosting of the many planned face-to-face activities. Originally an eleven-partner project backed by volunteer expert Advisory Board members, **CSI-COP** project successfully concluded with nine partners, produced an extra deliverable in the form of ‘ Guidelines for the EU on improving GDPR compliance’, and an industry award for the ‘ **Best Innovative Privacy Project**’ in the inaugural Piccaso Privacy awards in December 2022. The topic of the CSI-COP project concerned raising awareness of the rights to personal data protection accorded in the EU’s general data protection regulation (GDPR) among the general public. Additionally, CSI-COP aimed to informally educate volunteers providing them with skills as citizen scientists to join the project investigating the compliance of the GDPR. The investigations would focus specifically on the GDPR principles of ‘transparency’ and ‘informed consent’ in websites and app cookie notices, and in privacy policies.

It had been naively surmised that mobility restrictions would not affect such a project, since its main purpose entailed online computer-mediated tasks investigating compliance of the EU’s new regulation, the general data protection regulation (GDPR) by exploring how much of our screentime and online behaviour, using computers and smart mobile devices, is captured by third parties from websites we visit and apps we use. On the contrary, we found that COVID-19 restrictions around the globe restricting citizens’ interactions with families, friends and outdoor activities, detracted from time to spend on volunteering computer work. Using up precious time for more computing related effort could be seen as an extension of homeworking, which most people were required to do across 2020–2022. Here we present the effect of the corona virus, which emerged in late 2019, on CSI-COP activities from the first force majeure: pandemic *lockdowns* across the world with nations implementing stay-at-home restrictions from March 2020. Rearranging order of tasks, transforming planned face-to-face activities as online events, and deploying selected social media platforms ensured citizen science engagement remained on track, albeit with a reduced number from that envisaged during the proposal planning stage in late 2018 and early 2019. Regular project newsletters distributed widely and made accessible for online-reading in English from the project website, as well as translated in different languages by the partners, carried the project’s message to thousands across Europe and beyond ( CSI-COP Newsletters). The next sections detail the nature of the force majeure and the necessary changes to project tasks to address the pandemic effects in CSI-COP and how the partners overcame citizen science engagement challenges.

### The emergence of COVID-19 in late 2019

Now that the world is back to seemingly normal interaction, able to travel again and meet face to face, we remind of the force majeure that severely disrupted human activities for nearly three years. On 11 March 2020, the World Health Organisation (WHO) declared a corona respiratory disease as a world virus ( WHO, 2020b; Singh & Singh, 2020). WHO’s announcement was necessary due to the alacrity and efficiency of the corona virus causing debilitating symptoms including fever, sore throat, loss of smell, and breathing difficulty ( Singh & Singh, 2020). Severe symptoms led to hospitalisation and intensive care unit of some patients with further risk to their health following complications due to contracting the new corona disease in early 2020 ( Leung *et al.*, 2020). WHO announced the worrying spread of the disease around the world through a media briefing:

“WHO has been assessing this outbreak around the clock and we are deeply concerned both by the alarming levels of spread and severity, and by the alarming levels of inaction. We have therefore made the assessment that COVID-19 can be characterized as a pandemic.” ( WHO, 2020a).

The WHO (2022) recorded the global excess mortality associated with COVID-19 in the twenty-four months from January 2020 to December 2021 as 14.91 million. This number represents “9.49 million more deaths than those globally reported as directly attributable to COVID-19” ( WHO, 2022). In defining ‘excess mortality’, the WHO stated this is “the difference between the total number of deaths that have occurred and the number of deaths that would have been expected in the absence of the pandemic i.e., a no-COVID-19 scenario.” (2022). The WHO reported the corona pandemic’s “several waves” impacting different countries according to their “unique regional distributions, mortality levels and drivers” (2022):

“Twenty countries, representing approximately 50% of the global population, account for over 80% of the estimated global excess mortality for the January 2020 to December 2021 period. These countries are Brazil, Colombia, Egypt, Germany, India, Indonesia, the Islamic Republic of Iran, Italy, Mexico, Nigeria, Pakistan, Peru, the Philippines, Poland, the Russian Federation, South Africa, the United Kingdom of Great Britain and Northern Ireland, Turkey, Ukraine, and the United States of America (USA).”

CSI-COP’s Researchers spread across Europe and in Israel were also impacted by COVID-19 restrictions, the consequences were felt by female scientists and scientists with young dependents. Deryugina, Shurchkov and Stearns (2021) found that:

“All academics report substantial increases in childcare and housework burdens, but women experienced significantly larger increases than men. Female academics with children report a disproportionate reduction in research time, both relative to childless men and women and to male academics with children.”

The findings from different studies ( Blowers *et al.*, 2022; Deryugina *et al.*, 2021; Myers *et al.*, 2020) chime with the experiences of CSI-COP researchers across the severe pandemic years of 2020–2022. This included the effects of new and increasingly infectious variants of the original COVID-19 strain striking researchers leading to ill-health while trying to navigate the challenges of home-schooling, undertaking domestic duties and homeworking. The relevance of this paper is to serve as a guide of the “probability of occurrence of extreme epidemics”, being significantly less than in a hundred years’ time, the duration between the ‘Spanish flu’ (1918–1929) and COVID-19. Infectious diseases’ expert, Dr Nathalie MacDermott warns: “the next pandemic is around the corner - it might be two years, it could be 20 years” (in Sky News, 2024). Hence preparation and rapid response are crucial.

## Methods

### CSI-COP design

**Figure 1.**
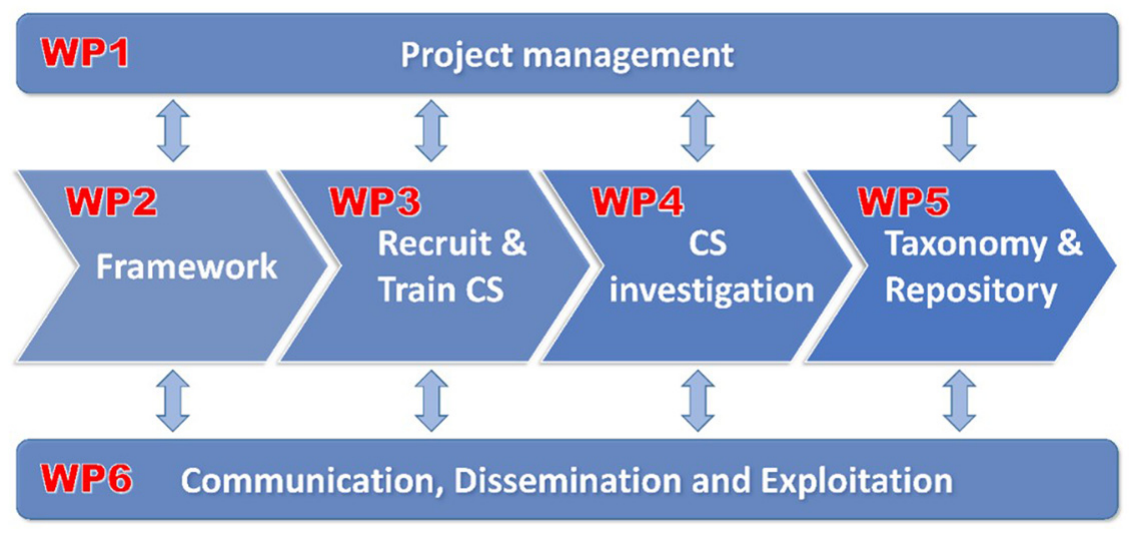
CSI-COP workflow diagram.

 CSI-COP research and innovation project was designed over six sub-projects, or work packages (WPs) to originally engage adults (over 18s) as citizen scientists ( Figure 1).

WP1: project management by the Coordinating partner – Coventry University, UK running the length of the project.
WP2: Framework for inclusive Citizen Science Engagement (research phase)
WP3: Recruitment and Training Citizen Scientists (citizen science engagement)
WP4: Citizen Science Investigating GDPR Compliance (citizen science engagement)
WP5: Open-access Knowledge Resource of Digital Trackers (innovation phase with citizen scientists contributions from WP4)
WP6: Communication, Dissemination and Exploitation (stakeholder and citizen science engagement)

From late March in 2020, team members in CSI-COP partner countries across Europe and in Israel were curtailed due to the pandemic lockdown restrictions in their countries, which included the academic partners having to adapt to required online teaching and learning new web tools made necessary due to closure of universities and suspension of classroom education. Additionally, CSI-COP consortium colleagues who were also parents had to take on teaching their children at home due to school closures. Thus, requirements of the expected outputs detailed in the project’s work programme necessitated innovative thinking rearranging the order and feasibility of certain activities and tasks’ delivery to mitigate some limitations. How and which social media platform to use was paramount in considering citizen’s data protection. This is explained next.

### CSI-COP and social media use

CSI-COP philosophy excluded the use of a major social media organisation: Facebook (now Meta) and its associated apps, Instagram and WhatsApp ( Holroyd, 2021). From the exposé of the Cambridge Analytica scandal, the world learnt how Facebook turned a blind eye allowing intrusions via its platform of users’ privacy capturing personal data without their consent. Facebook’s inaction led to democratic controversies. Cambridge Analytica’s data scientist Christopher Wylie revealed how easy it was to remotely observe someone’s Internet surfing from thousands of miles away without the surfer’s knowledge ( Wylie, 2019: p.53). Having received “unredacted, de-anonymised census” from the government of Trinidad and Tobago, and a successful request to Caribbean telcom companies “to ask if SCL [Cambridge Analytica’s parent company], could tap into their data ‘firehose’ in real time” Wylie divulged that “SCL was able to tap into the telecom firehose, pick an IP address, and then sit and watch what a person in Trinidad was browsing on the Internet at that very moment” ( Wylie, 2019: p.53). Wylie went on to disclose that the IP address was looked up and then Google Maps satellite view checked to “see the neighbourhood this person lived in” ( Wylie, 2019: p.53).

Facebook’s failure to protect its users’ from alleged manipulation and permitting third-party access to their data was known before the start of CSI-COP. During the project’s execution more information came to light pertaining to Facebook's inability to protect its users personal data from hackers: **more than 530million Facebook users’ personal data was found available on a website for hackers in April 2021** ( Holroyd, 2021). The hacking of personal information of 533million Facebook users were mostly users in these countries:

-    More than 35 million in Italy
-    Over 32 million in the US
-    Almost 20 million accounts in France
-    11 million users in the UK, and
-    6 million users in India.

Lomas (2021) reported that the data dump of information hacked from Facebook included these data points that could identify individuals:

-    Facebook IDs
-    Full names
-    Phone numbers
-    Locations
-    Birthdates
-    Bios, and
-    Some email addresses

Privacy advocate Max Schrems, founder of ‘none of your business’ (NOYB) has filed complaints holding big tech accountable for their data collection practices to ensure compliance of the GDPR. In a ruling by the Court of Justice of the European Union (CJEU: C-446/21: Schrems v. Meta), brought by Schrems, the CJEU “backed the lawsuit against Meta” to massively limit “the use of personal data for online advertisements” and to limit “the use of publicly available personal data to the originally intended purposes for publication” ( NOYB, 2024). NOYB’s campaigns help raise public awareness about privacy issues, the underlying mission of the CSI-COP project. The EU’s Digital Services Act (DSA), which came into force in February 2024, after the conclusion of the CSI-COP project, not only “complements the rules of the GDPR to ensure the highest level of data protection” ( EC, 2024) it also acts as a democratisation force alongside regulating online technologies since social media platforms exert a lot of power and have great impact from the collection of their users’ data. Hence the GDPR can be seen as a democratic topic.

CSI-COP’s privacy-by-default approach to protecting citizen scientists' personal information while investigating GDPR compliance effected the decision to limit use of social media platforms, especially Facebook, in project communication and dissemination. CSI-COP’s strategy aimed to a) limit tracking of CSI-COP citizen scientists and researchers as they performed online activities for the project; b) investigate GDPR compliance in websites and apps that citizen scientists and the project researchers used, and c) learn to what extent limiting social media use affected general public engagement. With an acceptance that social media cannot be totally set aside for engagement, the project created CSI-COP LinkedIn and Twitter/X accounts to communicate opportunities for volunteer participation in the project. Alongside these two social media platforms, deploying the consortium’s marketing efforts through partners’ resources, and team members endeavours, CSI-COP disseminated activity news encouraging participation. Additionally, project partners could use their own organisation’s various social media accounts, or their individual ones (e.g., Snapchat, Facebook, TikTok) to raise awareness of their involvement in the project, promote their activities and engage interested member of the public.

### GDPR compliant project website design

Some Internet access issues affected home working in the early days the project during COVID-19 lockdown. This did provide an opportunity to research ethical issues in creating a GDPR compliant project website. A month’s delay in launching the project website, from Month 3 (March 2020) to Month 4 (April 2020), realised a GDPR compliant design. CSI-COP innovated a no-tracking project website at csi-cop.eu. Reverse-engineering a popular web development platform (WORDPRESS) extracted out third-party trackers already embedded in the development environment leaving a privacy-by-design website ( Figure 2, Table 1). This feature is signalled to CSI-COP website visitors through transparent text in the cookie banner at the bottom of each page on the site.

**Table 1.** Images of CSI-COP’s GDPR compliance website.

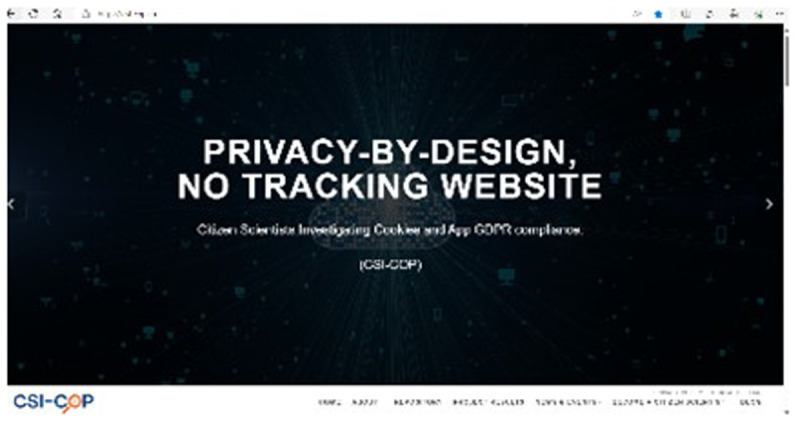	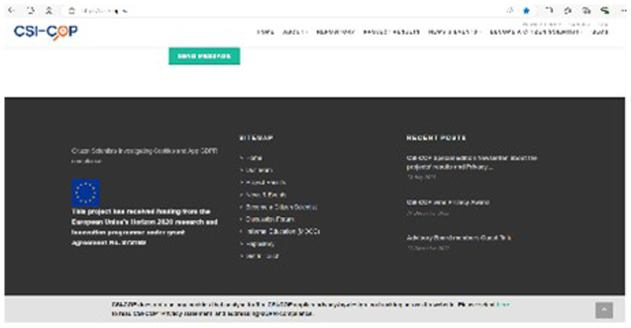	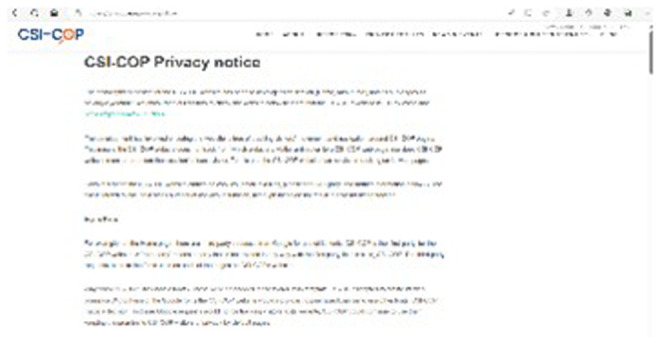
**Figure 2: Top of CSI-COP website home ** **page announcing the site as ‘privacy by ** **design’: https://csi-cop.eu/**	**Figure 3: Bottom of CSI-COP website home page ** **declaring no analytical cookies used across the ** **site: https://csi-cop.eu/**	**Figure 4: CSI-COP Privacy notice: https://csi-cop.eu/privacy-policy/**

“ **CSI-COP does not use any cookies that analyse traffic. CSI-COP applies privacy-by-design, no tracking across its website**.” ( Figure 3 in Table 1).

The website’s privacy notice explaining the project’s no tracking philosophy was crafted in simple to understand, transparent language conveying the project’s commitment to not only investigate GDPR compliance, but also to comply with the regulation’s principles of informed consent and purpose limitation ( Figure 4, Table 1). Images from CSI-COP’s website in Table 1 illustrate the privacy by design commitment:

**“**Please select here to read CSI-COP ‘Privacy statement’ and addressing GDPR-compliance.” ( Figure 4 in Table 1). **”**

Collection of data is limited in CSI-COP’s website. Apart from emails collected from interested parties registering on the website, no other personal information is collected from csi-cop.eu. Individuals could register without using their real name. The email would be exclusively used to update registered individuals with project news and invitations to participate in, or attend events. The website main menu featured pages, including information on becoming a CSI-COP citizen scientist. Link to project results could be accessed from the main menu, as well as newsletters which could be read online or downloaded as reports, blog posts, and an FAQ. The website is also the online location that houses the project’s innovation: the searchable Repository of investigated websites and apps: https://csi-cop.eu/repository/ discussed later in this paper.

### Ethical approval

Alongside developing the project website, during the course of CSI-COP the Coordinating partner (Coventry University) applied for ethical approval from its university research ethics approval committee. Three ethics applications were made to encapsulate phases and change, for example, to include under-18s in the latter stage of the project. In the first phase featuring desk-top research, ethical approval was sought to proceed without any external participants and volunteers. Ethics approval was granted for this first stage of the project under Coventry University’s CU Ethics process and was authorised with certificate issued under reference P109200 in September 2020. In 2021 the Coordinating partner applied to gain fresh ethical approval through updating Coventry University’s research ethics committee about an extension sought from the EU for CSI-COP due to COVID-19. This second ethics application to Coventry University’s research ethics committee submitted GDPR-compliant documentation for the transparent engagement of citizen scientists by informed consent. The change was approved in August 2021 under ethics certificate P125708. A final ethical application was made to Coventry University’s research ethics committee for the purpose of expanding engagement to include Under-18s in CSI-COP. New transparent documents were created to comply with the GDPR to gain written informed consent from parents or guardians of Under-18s for their participation in CSI-COP and learning about any tracking in children’s or other apps they might use on their personal devices. Approval was granted in August 2022 through ethics certificate P139906. All participants were fully informed about the purpose of the project through GDPR-compliant Participant Information Sheets which were crafted in English and then translated by the CSI-COP partners for their local participants. Written consent was gained from citizen scientists through GDPR-compliant Informed Consent sheets. Written consent was also gained from Parents or Guardians of Under-18 citizen scientists. Participant Information Sheets and Informed Consent Sheets are accessible from the CSI-COP Zenodo repository ( Zenodo, nd).

In all aspects of CSI-COP, responsible research was conducted with full and proper consideration given to any possible ethical issues that could occur. Hence three ethics applications submitted during the life of CSI-COP project, which were approved by the Coordinating partner, Coventry University’s research ethics committee. All identified risks were addressed, eradicated, or mitigated. Benefits of CSI-COP participation were clearly demonstrated through new knowledge and practical skills gained by the volunteer citizen scientists. The new knowledge created during the project, around the extent of online tracking and the status of GDPR compliance in websites and apps investigated in CSI-COP, is searchable through the project’s innovated Repository.

## Overcoming COVID-19 challenges Project research phase

As a research and innovation project, CSI-COP’s research began with understanding ethical engagement of the general public as collaborators in scientific endeavours. This phase aimed to produce a framework for engaging citizen scientists as part of work package 2 activities ( Figure 1), consequently a rethink was necessary. There was no playbook for citizen science during a pandemic. Nonetheless, CSI-COP began its research phase mostly unaffected by the mobility restrictions since the actions involved in WP2 were performing desk-based research exploring citizen science methodology and creating a living data management plan (DMP). For the latter, CSI-COP’s first DMP ( Shah *et al.*, 2020) did not entail gathering or processing any personal data. However, data management was an evolving process with two further iterations during the project reflecting the later stages engaging the general public and the inclusion of citizen scientists.

CSI-COP’s framework design leveraged the ten principles from the European Citizen Science Association ( ECSA, 2015). Multidisciplinary research conducted by the partners produced two reports gaining insights into i) best practices in inclusive citizen science in the ‘ CS Research Report: Public report on current methods in CS engagement’ ( Ignat *et al.*, 2020), and ii) optimum methods to engage a diverse cohort in a report ‘ Guidelines for Diverse Citizen Science Recruitment’ ( Hinsenkamp *et al.*, 2020). These two reports fed into a third missive detailing the specific framework CSI-COP adopted with recommendations from Ignat *et al.* (2020). The framework was based around the informal education and experiential learning that CSI-COP would provide to engaged members of the public. This entailed increasing the scientific literacy around citizens’ rights accorded in the GDPR and the provision of practical training to improve management of personal data and privacy online ( Stepankova *et al.*, 2020). The pandemic restrictions did not affect the delivery dates for the project’s research results: the three research reports were completed within the original deadline for CSI-COP’s research work package (WP2 in Month 9–September 2020). CSI-COP’s framework also realised a database of diverse organisations with partners identifying civic, research and other organisations across Europe to contact in relation to raising awareness of CSI-COP, its mission, collaboration and participation opportunities among their members. The ‘Dataset of Organisations to Approach in Citizen Science Projects’ ( Shah & Winter, 2022), was made available through Zenodo open access platform: https://zenodo.org/records/6780048

CSI-COP partners utilised that resource directly approaching the identified stakeholders by telephone, through website online contact forms, and where available, through email communication raising awareness of the project’s activities and opportunities for members of these organisations to get involved and become citizen scientists. The benefits disseminated were access to free informal education and training experience equipping people with the knowledge to better protect theirs and their family members’ personal data online. Through these benefits adult members of the general public had an opportunity to act as citizen scientists volunteering alongside project partners together investigating the extent of online tracking and whether the GDPR was being complied with in websites and apps.

### Free informal education – MOOC and validation

**Figure 5.**
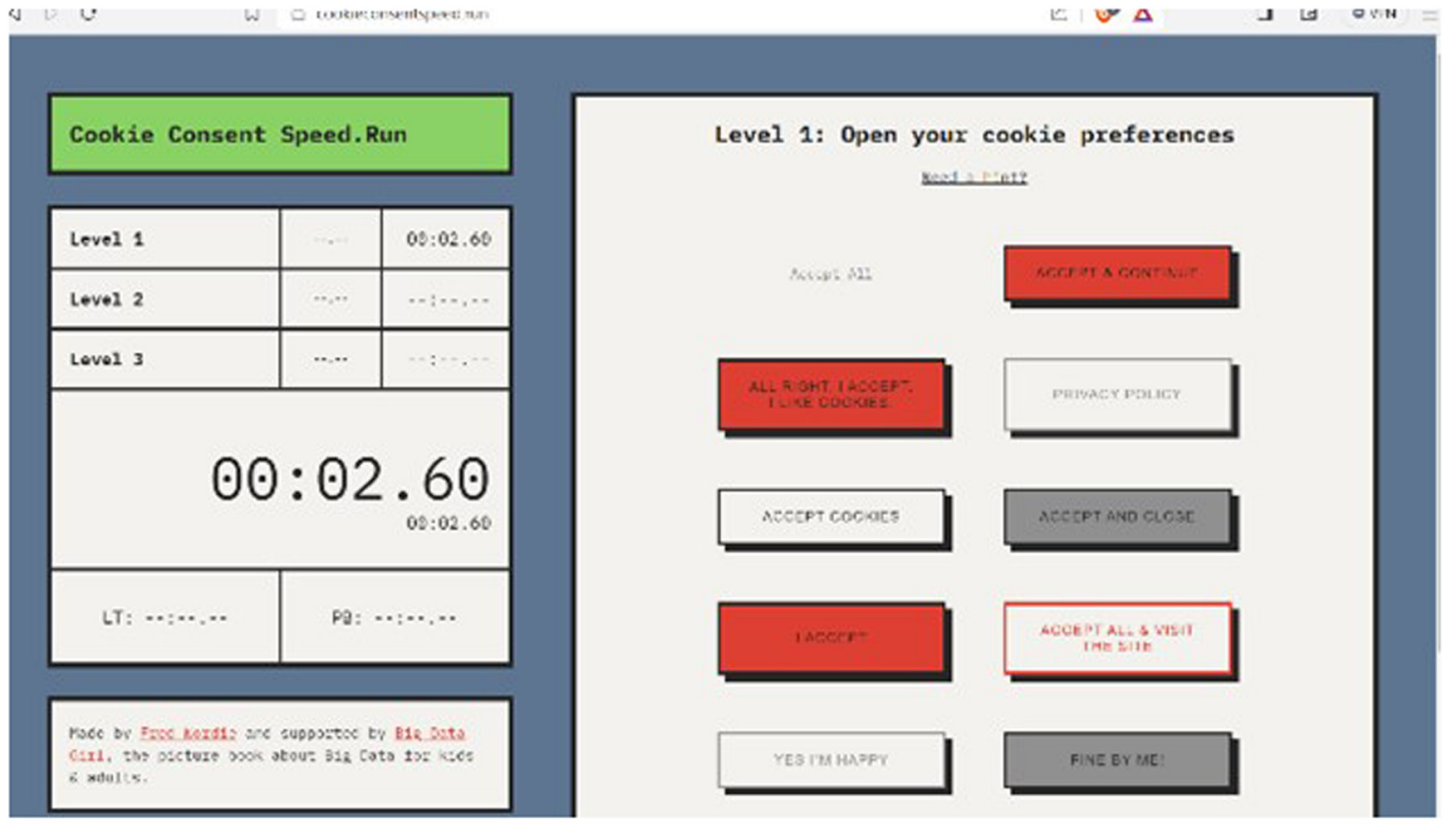
Cookie Consent Speed.Run play.

CSI-COP’s original work plan involved recruiting citizen scientists from the 7 ^th^ month in the project (July 2020), as part of the work package on ‘Recruitment and Training Citizen Scientists’ (WP3). Due to the pandemic effects, a two-month delay was requested from the EU for the start of WP3 and to reorder its two tasks. This allowed time to focus on innovating an interactive free online informal education resource as a short ‘massive open online course’ (MOOC) before engaging the general public. The time was used to create meaningful and practical exercises) ( Mentimeter (n.d.), along the course affording learners to take stock of the informal lessons. The internet was also scoured to find complementary exercises bringing the lessons alive and relevant to real-world data protection and privacy issues. A free online game was found, a fun-while learning about necessary and tracking cookies: Cookie Consent Speed.Run which CSI-COP project researchers played for relevance. The first author’s [HS] play is shown in Figure 5.

Players in the cookies consent run game are timed across its three levels accepting only necessary cookies while rejecting unnecessary ones. Fred Wordie, creator of the cookie consent run game, used real cookie banner text from across the Internet to “explore how we as a society engage with technology and each other”. Wordie’s cookies consent run game demonstrates the ambiguity of many website cookie notices and cookie banners that lack transparency in gaining informed consent for non-functional cookies that might be tracking website visitors and passing data on to third parties.

CSI-COP sub-contractor ( Pat Walshe of Privacy Matters) co-created the MOOC ‘Human right to privacy online’ in English, and as part of designing interactive exercises raised awareness of free online privacy audit tools, including webbkoll and PageXray for websites, and Exodus Privacy for apps. These tools present a breakdown of what lies beneath a website, or an app. For example, using webbkoll to check beneath the UK’s BBC website reveals that it allows “165 third-party requests to 21 unique hosts”. Without their knowledge, BBC visitors’ information requests are sent to advertising companies, including Amazon and Google. Utilising such free online privacy audit tools ( webbkoll; Exodus Privacy) provided MOOC learners with experience and discovery of the extent to which we are tracked online without our knowledge, especially when cookie notices do not make it transparent who has access to data about their visiting a website, or using an app.

**Figure 6.**
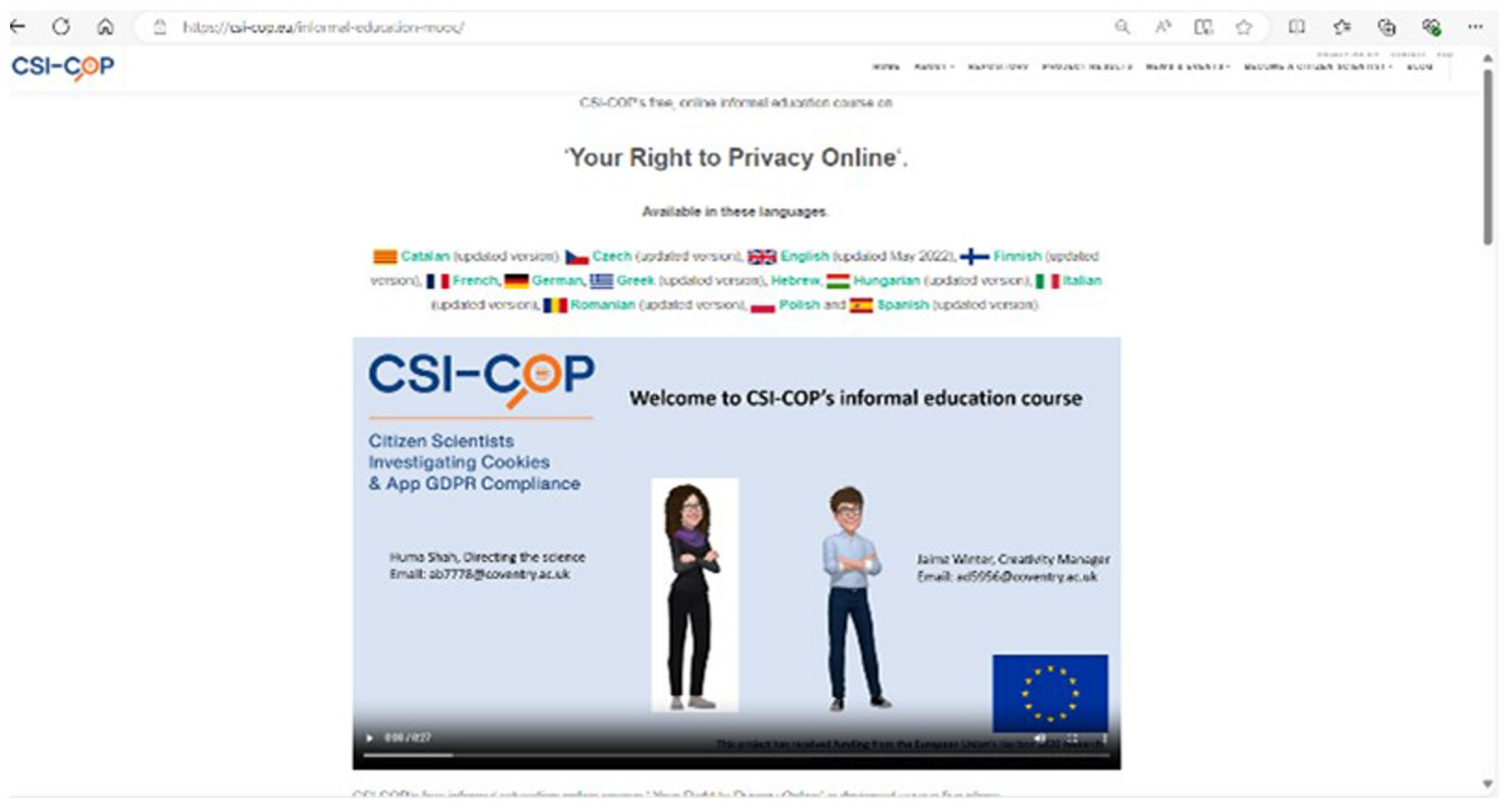
CSI-COP MOOC page.

On the suggestion of the EU’s appointed external Reviewer during the project’s first period review, the MOOC title was changed to ‘ **Your Right to Privacy Online**’. With a brief self-assessment ‘review’ after each of the five steps in the MOOC, a test of ten multiple choice questions concluded the CSI-COP’s MOOC resource. A CSI-COP certificate on completion could be claimed on completing the course and submitting the test answers to the local project partner, or to the coordinating partner. Validation of the English language MOOC was conducted internally and externally. External validation was gained by inviting students and the general public to try out the MOOC and feedback improvements or issues. The MOOC’s internal validation was conducted by CSI-COP legal partner – STELAR. Following this the English language MOOC was made available for reading online or downloading as a document from CSI-COP’s website ( Figure 6).

Partners translated the MOOC making it accessible in different languages (including Czech, Greek, Spanish, French, Hungarian, Romanian, and in Hebrew). The English language MOOC was also transformed into units for completion by visitors completing it on the EU-Citizen.Science platform. 

### Overcoming challenges to recruiting citizen scientists during COVID-19

Before the project start, it was envisaged that thousands of individuals would complete the MOOC since people were confined to the home, and homeworking. As reported by Sotgiu and Dobler (2020): “the rigidity of lockdown measures radically changed social interactions with virtual meetings replacing face-to-face meetings to reduce the risk of transmitting the corona virus”. Hence the provision of informal learning from the planned face-to-face events moving to online meetups was a clear option, rather than abandoning the idea of including the public. In higher education, the impact of moving learning from class-based to online varied from student to student. In a short poll of first year undergraduates in the lead partner’s university module ‘Artificial Intelligence, Creativity and Ethics’ showed online learning suited those students who lived far from campus saving them commute time ( Box 1). Other students who suffered “anxiety” and felt a class setting was “uncomfortable” believed the online format made “learning far harder” ( Box 1).

Box 1 **Anonymised comments from Coventry University first year students during pandemic-enforced online learning**

1. Easier for people who can't commute
2. It can be perfect for a lot of certain individuals that have anxiety and also for anyone with travelling problems. However online learning has a lot of disadvantages.
3. Its ok for people that don't feel comfortable in a class setting, but isn't ideal for the most part as it makes learning far harder.
4. I can see benefits of online learning such as: allowing students to learn from anywhere they are at, being able to send over a large amount of information about the course for students to access anytime and it can allow for more convenient extra help sessions as it can be done anytime without traveling.

Mindful of the effect of online sessions on different individuals’ learning Engzell *et al.* (2021), as already explained, CSI-COP’s free short informal learning resource, the MOOC, was packed with interactivity, web links to follow up for more online material on data protection, and after translations, accessible in partner local languages. CSI-COP partners’ efforts in communication and dissemination spread the word about the availability of the project’s MOOC: over six hundred completions were recorded. This number was expected to be greater but another major challenge handicapped CSI-COP: two university partners suffered cyberattacks. This especially affected UAB’s general public engagement: they faced a ransomware attack in which cybercriminals gained access to the university's systems through phishing, using stolen credentials from a standard user. The attackers exploited vulnerabilities to access servers, analysed the network, and attempted to block data and disable backups. With the help of Dell and Fortinet, the UAB successfully recovered from the attack without paying the ransom. Key measures included creating a clean parallel system and completing a forensic analysis before resuming operations ( ARA, 2021). This incident highlighted the importance of cybersecurity and served as an opportunity for the university to modernise its systems. The UAB emphasised the ethical and practical reasons for refusing to negotiate with the attackers, avoiding further targeting by criminal groups.

These cyberattacks on partners during CSI-COP’s operation reinforced the practice of limiting the collection of participants’ personal data and being vigilant to cyber threats, including attacks against the project’s website. Personal data protection, online privacy, and tracking of children through apps drew interest in CSI-COP’s topic, some of that interest progressed individuals to complete the MOOC in one of the available languages. There was less desire to become volunteer web and app investigators as citizen scientists. Responses from members of the public declining participation included: “The CSI-COP topic is very interesting. But I am afraid I am too busy to spare some time for participation.” Nonetheless, one-hundred and ninety-one individuals, from the over six hundred who completed the MOOC, acted as citizen scientists contributing investigations of websites and apps they were familiar with. The next section presents rolling out the MOOC during COVID-19 challenges.

### Overcoming effect of COVID-19

Citizen science is, by its nature, a participatory activity democratising science. Original plans for public engagement preferred adults to gain research ethics approval. The lead partner completed a research ethics application for the whole project requiring all partners comply with the proposed actions in an ethical manner to engender trust with the community of citizen scientists recruited to contribute to CSI-COP. Questions in the ethics application included providing an outline of the project’s “principle methods” and whether data would be collected “off campus” ( Box 2). Responsible research was evidenced through CSI-COP’s declaration in the ethics application that project activities included “equipping citizen scientists with the skills to evaluate human rights in the digital age”, and that recruitment of the public would be “inclusive from a diverse range of the public” ( Box 2). Ethical approval was gained from the lead partner’s research ethics panel (under reference P109200).

Box 2. **Coventry University’s ethics application for CSI-COP activities**

**Table:** 

Ethics application question	Lead Partner response for CSI-COP consortium
What is the purpose of the project?	Leverage citizen science methodology to investigate compliance of the general data protection regulation (GDPR) through cookies and apps embedded in websites and in smart phone apps.
What are the planned or desired outcomes?	1. Setting up the CSI-COP project citizen science initiative to generate new knowledge and understanding of the extent of tracking online. 2. Informally educate CSI-COP cohort recruited from the general public to act as citizen scientists in the project. 3. Equip CSI-COP citizen scientists with the skills to evaluate human rights in the digital age 4. Create a taxonomy of different types of online trackers made available as an accessible repository of cookies in websites and apps.
Outline the principal methods you will use	1. Ethical and inclusive recruitment from a diverse range (gender, geographical, socio-economic) of the general public and beyond using a variety of organisations (Teachers; Parents; Women groups, privacy and security organisations, data protection activists, tech journalists, ethicists, policy-makers, GDPR experts, privacy lawyers) 2. Free-to-attend informal education workshops and MOOC equipping citizen scientists with the knowledge of their rights accorded in the GDPR and how to explore and find different types of cookies (session/persistent; first-party/third-party) beneath websites and in apps 3. Remote online investigations 4. Supporting and mentoring CSI-COP citizen scientists creating privacy champions continually motivating through interaction via a privacy-by-default CSI-COP project website 5. Co-creating a taxonomy of different types of trackers 6. Co-innovating a repository of online trackers 7. Collaborate with sister citizen science projects funded by the EU and others 8. Communicate project purpose to build partnerships promoting privacy-by-design development of websites and apps. 9. Disseminate and exploit CSI-COP findings on the extent of tracking online 10. Devise CSI-COP certification for privacy champions recognised by organisations as skills in data protection 11. Organise a series of events, inclusing parent-teacher roundtables, Stakeholder cafes, and main project event, including with policy-makers in Brussels 12.Create project legacy working group to promote privacy-by-default beyond the project life turning the tide of ubiquitous tracking online.
Does any part of the project require data collection off campus?	All the investigations of cookies on Internet websites and in smart phone apps will be conducted online. All the data to be collected will not be from any work conducted 'in field' or 'in communities'.

On the suggestion of one of CSI-COP’s Advisory Board members, to include school pupils and spread the word up to parents and across to family members, the ethics application was revised to engage school pupils aged 14–17 expanding the reach of the project and to upskill parents and schoolteachers. Another Advisory Board member put CSI-COP in touch with the **International Teacher Magazin**e realising a short article ‘ The Cookie Jar: Protecting Children’s Data’ ( Shah, 2021).

Despite COVID-19 effects, alongside project management (WP1) running from the first month in the project (January 2020), CSI-COP’s research phase, WP2, concluded successfully and on time at the end of the project’s first nine months (January-September 2020). CSI-COP’s citizen science and recruitment phase benefited from opportunities to interact online with other recipients of the EU Horizon2020 SwafS award. Through attended monthly meetings between 2020 through to the end of 2022, CSI-COP learned from other funded projects that some were concerned with the ‘citizen science process’, rather than actively engaging members of the public, for example MICS ( MICS.tools, n.d.). Reaching members of the public and engaging them appeared more successful for citizen science projects concerned with nature, climate, sustainability and health, including mental health. Adapting the ‘ Big Plastic Count’ household plastic waste initiative, CSI-COP Partners also collaborated to organise an online ‘ **Big Cookie Count**’ event in May 2022. This online event organised as an inclusive Zoom webinar hosted separate digital rooms offering partners communicating in different languages: Catalonian, Czech, Finnish, Greek, Hebrew, Hungarian, Italian, Polish, Romanian, Spanish and Turkish. Partners also offered specialist topic rooms for interested individuals to join a CSI-COP partner for a chat: one for cybersecurity, and one for data-privacy. CSI-COP’s ‘Big Cookie Count’ boosted reach and gained interest in the topic and a desire from individuals to improve their online personal data protection with the knowledge gained in this event. This special CSI-COP event was reported in the project’s 7 ^th^ Newsletter (August 2022). 

As explained earlier in this paper, it was assumed that, since people were working from home during the pandemic, including using Internet-enabled technologies, there would be great interest from the general public to explore beneath websites and apps to find what third-party tracking lurked beneath. In contrast, CSI-COP partners found engagement of the general public and recruitment of citizen scientists difficult during the pandemic. The continuing effects of COVID-19 lockdowns, and restrictions to in-person meetings and leisure activities in 2021 entailed CSI-COP seeking extensions to the project through two Amendments to the grant agreement. With the termination of one university and one SME partner, the EU granted extensions to CSI-COP from June 2022 to August 2023. Approved changes and project extension involved a redistribution of terminated partners’ tasks and extended deadlines for task outcomes. Two smaller partners took on extra work, after the termination of a university partner, contributing to recruiting citizen scientists: the Association of Hungarian Women in Science (NaTE), and Immer Besser for Hungarian and Romanian speakers respectively.

CSI-COP partners applied local knowledge in resolving community-specific pandemic-related challenges to reach their public. Engagement methods included a successful ‘personal touch’ approach telephoning organisations directly explaining CSI-COP purpose and engaging members. Concomitant activities included email approaches to stakeholders and organisations categorised in CSI-COP’s WP2 dataset of identified organisations. Partners also used the ‘Contact us’ forms on organisation’s websites to raise awareness of the project’s objectives and participation opportunities. Once mobility restrictions were lifted from the summer of 2022, to make up for loss of reach during pandemic lockdown, in-person events were organised in public places, leveraging the European Researcher’s Night, and also hosting tents in big events such as festivals (Ideas Fest, UK; Sziget Festival, Hungary). Over 20 workshops were organised using the MOOC across Europe and in Israel as either online, in-person or hybrid events. Face-to-face workshops were organised in London, Coventry, Athens, Patras, Budapest, Tel Aviv, Prague and in Cluj Napoca (Romania). Communication and dissemination activities, including through a regular project Newsletter featuring guest articles, and CSI-COP citizen scientists’ interviews, built a thriving community of Privacy champions. 

### Citizen science activities

Over 630 members of the public ( Table 2) across Europe and in Israel completed the free educational course ‘Your Right to Privacy Online’ ( Shah *et al.*, 2022a; Shah *et al.*, 2022b). Of the those who completed CSI-COP MOOC, 191 individuals joined CSI-COP as citizen scientists ( Table 2).

**Table 2.** Number of CSI-COP citizen scientists.

CSI-COP Partner	MOOC Completions Numbers (number of individuals who completed CSI-COP MOOC)	MOOC language Completions	Number of citizen scientists who contributed investigations
CU (Coordinator)	78	77 in English + 1 in French	34
UPAT	315	Greek	71
UOULU	11	10 in Finnish + 1 in English	11
BIU	71	Hebrew	23
CTU	81	Czech and English	34
UAB	28	Spanish + 1 in Catalan	1
NaTE	24	Hungarian	9
IB	23	Romanian + 3 in English	8
**TOTAL**	**631**		**191**

One reason given for not progressing from MOOC completion to becoming a citizen scientist included other commitments preventing people from going further as volunteers. Individuals informed them having to take on extra tasks in their own jobs, due to their having to take on the work of colleagues off-sick with COVID-19. Others advised that they were not interested in investing time to investigate their internet usage. Non-attending registered participants were sent the MOOC, or the link to access the MOOC from CSI-COP website to encourage people to take advantage of the free course and learn about how we might be tracked online in websites we visit and apps we use. CSI-COP’s citizen scientists were trained through one-to-one walkthroughs with a local partner in a local language in an online session following their MOOC completion and giving their informed consent to join as volunteer GDPR -compliance investigators. A bespoke recording tool was created as an MS Excel spreadsheet to capture findings of the number and type of cookies found in websites and apps following investigations (see Taxonomy of Digital Cookies and Online Trackers: Shah *et al.*, 2023).

**Figure 7.**
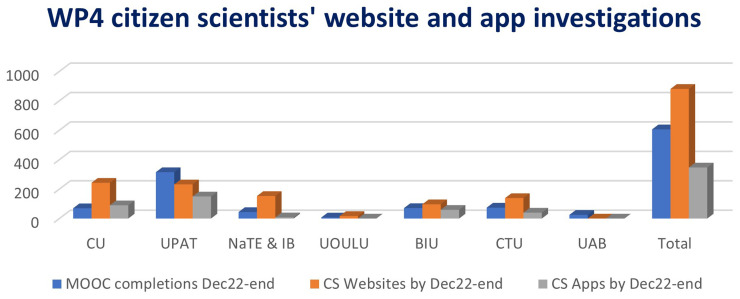
CSI-COP citizen scientists MOOC completions and website and app investigations.

The two smaller partners (NaTE and IB) between them realised 47 MOOC completions and engaged 17 citizen scientists. Due to unforeseen circumstances explained earlier (cyberattacks) there was a delay to the start of citizen science engagement in two other partners. A surprise was the low interest in engagement from Finland. The partner in this country (UOULU) gained 11 MOOC completions, all progressing to citizen scientists ( Table 2). The MOOC completions and number of citizen scientists who joined the project were lower than CSI-COP expected to engage before the start of the pandemic in the proposal planning and grant agreement completion phase. As Singh and Singh (2020) reported on lockdowns, the pandemic related restrictions “created an environment of fear, anxiety and stress among the developed and developing societies.”. The project’s topic, the extent of online tracking through third-party cookies on websites and in apps, is a topic that clearly engaged some with angry questions such as, ‘Why is the government not doing anything about all this tracking?’. Nonetheless, between the 191 citizen scientists, their invaluable contributions together produced over 1350 investigations: over 1000 websites and over 350 apps ( Figure 7).

As explained, CSI-COP gave itself challenges by avoiding the creation of pages on Facebook or videos on YouTube, which could have increased engagement. These two platforms’ owners (Meta; Google) are frequently at the centre of privacy intrusion and data breaches ( Google anti-trust, 2021; Holroyd, 2021). Nonetheless, CSI-COP partner organisations were not discouraged from using their existing Facebook or other social media platforms to engage their local public. UAB partner created their own Twitter account in Spanish posting information about the MOOC and project activities. CSI-COP used its LinkedIn account linking to newsletters, workshops and related information. CSI-COP do accept that, since both Facebook and YouTube are widely used platforms, the project naturally reduced its reach by keeping to its philosophy of attempting to protect the data and preserve the privacy of Internet users.

Nonetheless CSI-COP’s adoption of ‘ **reverse mentoring**’ engaging school pupils to upskill the adults in their lives (parents and adult relatives) reached under18s through schoolteacher-pupil activities. CSI-COP legal partner, STELAR, provided the legal expertise to meet the extra GDPR compliant threshold of involving under18s. Revised participant information documents were created to meet the ethics requirements to involve younger people in CSI-COP. STELAR also ensured secure data management was practiced by all partners through expert guidance and ensured compliance with the GDPR principles concerned with child data protection. Involving under-18s in CSI-COP activities raised awareness among about the extent of online tracking, for example, beneath healthcare and games apps targeted at children.

Anonymised analysis on *who were* CSI-COP’s citizen scientists was reported in ‘ Age, Gender, Socio-Economic and Geographical (AGSEG) Distribution Report’ ( Rigler *et al.*, 2023), in PLOS One journal article ‘ Modeling intrinsic factors of inclusive engagement in citizen science: Insights from the participants’ survey analysis of CSI-COP’ ( Hadad *et al.*, 2023). Article ‘ Beneath the bonnet of online privacy' was published by the EU Researcher journal in their August 2023 issue.

## Conclusion and further work

The corona virus pandemic certainly limited the awareness-raising of the CSI-COP project through nation lockdowns, limiting travel and necessitating working and learning from home. Challenges to engaging people in such a climate were added to by individuals who did not want to know how much they were being tracked online. Additionally, CSI-COP specific online activities did not entail enjoying nature through sports and outdoor play, actions which were disallowed during the pandemic restrictions. CSI-COP resolved force majeure challenges through proactive adjustments to the severe changes by creating a free interactive informal learning resource (MOOC), translating it into multiple languages for learning in own-time; adapting Greenpeace’s ‘Big Plastic Count’ for an online ‘Big Cookie Count’ event and transforming the MOOC into free-to-attend webinars organised by partners in their local language. 

CSI-COP demonstrated that it not only investigated compliance of the GDPR in websites and apps, but the project also set in place GDPR compliant processes to build trust with its engaged public and citizen scientists. The project innovated a searchable Repository of the websites and apps investigated by CSI-COP’s citizen scientists and researchers. Two policy briefs were produced to assist the EU with monitoring GDPR compliance. An extra deliverable generated a roadmap to guide the EU on strengthening the ‘legitimate interest’ feature which allows third-parties to gather personal data without clear informed consent. The societal impact from the project included raising public knowledge about a new regulation and encouraging citizens in Europe and beyond to participate in the scientific process by joining in observing and gathering data. The change in behaviour among the citizen scientists included applying their new understanding of the rights to data protection accorded in the EU’s GDPR, and their new practical skills, to better protect theirs and their young family members’ online privacy.

The value and benefit of citizen science, and the contributions from an informed, scientifically literate public to the production of new knowledge (extent of online tracking and compliance of the GDPR in the case of CSI-COP), was evident through receiving an industry award ‘Best Innovative Privacy Project’ in the inaugural PICCASO Privacy awards. Analysis of the number and types of website and app trackers enfolded in the searchable repository, and the extent to which the GDPR is complied with, is underway and will be reported in future peer-reviewed and other publications.

## Ethics and consent

In all aspects of CSI-COP responsible research was conducted with full and proper consideration given to any possible ethical issues that could occur. Hence three ethics applications submitted during the life of CSI-COP project which were approved by the Coordinating partner, Coventry University’s research ethics committee. All identified risks were addressed, eradicated, or mitigated. Benefits of CSI-COP participation were clearly demonstrated through new knowledge and practical skills gained by the volunteer citizen scientists. The new knowledge created during the project, around the extent of online tracking and the status of GDPR compliance in websites and apps investigated in CSI-COP, is searchable through the project’s innovated Repository.

## Data Availability

Data realised from the actions of the CSI-COP project are available from three open Repositories: **EU Cordis CSI-COP Results** page here: Citizen Scientists Investigating Cookies and App GDPR compliance | CSI-COP | Project | Results | H2020 | CORDIS | European Commission **CSI-COP project website** Results page here: Project Results – csi-cop **Open-access community platform Zenodo**: Zenodo **PLOS ONE journal article: Modeling intrinsic factors of inclusive engagement in citizen science: Insights from the participants’ survey analysis of CSI-COP | PLOS ONE** Zenodo: “ EU HORIZON2020 CSI-COP Anonymous dataset on Citizen scientists”. DOI: https://zenodo.org/records/10043687 ( Shah (2023).); “CSI-COP Dataset of Organisations to Approach in Citizen Science Projects”. Doi: https://zenodo.org/records/6780048 ( Shah & Winter (2022)).; “CSI-COP Citizen Science Participant Information Sheet” DOI: https://doi.org/10.5281/zenodo.14713029 ( Shah (2025a).); CSI-COP GDPR-compliant Participant Information Sheet: Under-18s DOI: https://doi.org/10.5281/zenodo.14795186 ( Shah (2025c).) and CSI-COP GDPR-compliant Informed Consent Sheet for Under-18 Parent or Guardian., DOI: https://doi.org/10.5281/zenodo.14795668 ( Shah (2025b).) This project contains the following extended data: a. CSI-COP Anonymous CS dataset_D4.3.xlsx https://zenodo.org/records/10043687 b. **CSI-COP Deliverable D2.3 - Organisation Dataset_290622.pdf Projects** https://zenodo.org/records/6780048 c. **CS Volunteer Information Sheet.pdf** (for informed consent) https://doi.org/10.5281/zenodo.14713029. d. Parent or Guardian Information Sheet for Participation of Under-18s https://zenodo.org/records/14795186 e. Parent or Guardian Informed Consent sheet for Participation of Under-18s https://zenodo.org/records/14795668 Data are available under the terms of the Creative Commons Attribution 4.0 International
