# Supplementary material for: Force majeure impact on citizen science: Perspective from an EU funded project
Source: Open Res Eur. 2025 Mar 3;5:59. [Version 1] doi: 10.12688/openreseurope.19184.1 (PMC11986408; doi:10.12688/openreseurope.19184.1)
Supplement: Peer review history

# Reviewer report: Reviewer response for version 1

**DOI:** 10.21956/openreseurope.20763.r52276

**Published:** 2025-04-10

**Authors:** Cosmos Nike Nwedu

**Affiliations:** 1 Faculty of Law, Department of Public and Private Law, Alex Ekwueme Federal University Ndufu-Alike (Ringgold ID: 439477), Ikwo, Ebonyi, Nigeria; 2 Kampala, Kampala International University, Kampala, Central Region, Uganda

Thank you for the opportunity to review the article entitled “Force majeure impact on citizen science: Perspective from an EU funded project”. The article addresses a significant topic of organisational and societal relevance by analysing challenges and solutions to conducting citizen science and research innovation activities during a pandemic. More specifically, it focuses on an EU-funded international project concerning GDPR compliance that occurred during the COVID-19 pandemic, which started in late 2019.

 While the article is generally well-structured, clearly written, and offers some original insights, I have the following comments and/or suggestions for the authors.

 1. The final line of your abstract below is muddled with the use of the word "rethought". Please paraphrase for more clarity.

 “Overcoming challenges imposed under force majeure conditions, a citizen science project funded under the EU Horizon2020 research and innovation scheme SwafS15-2019, rethought experiential learning through volunteering screen time”. 

 2. There are different Sections and/or Subsections in the article that are currently unnumbered. I believe that appropriately numbering them will enhance the clarity of its structural design.

 3. You made the following claim in the last paragraph of your “Introduction”:

 “… we found that COVID-19 restrictions around the globe restricting citizens’ interactions with families, friends and outdoor activities, detracted from time to spend on volunteering computer work”.

 How does the above claim imply that individuals who volunteer online cannot do so from their homes, assuming they have personal computers available? Please substantiate your claim with more explanation.

 4. Although the quality of your language is generally satisfactory, there are a few instances where significant grammatical errors occurred and non-plain languages used. These must be addressed. For example, the first line/paragraph of the Section titled “ **GDPR compliant project website design**” reads as follows:

 “Some Internet access issues affected home working in the early days the project during … “.

 The paragraph above tends to have a grammar issue that needs to be corrected. In other words, a preposition is missing there. More so, it is essential to replace the word “chime” in the first line of the last paragraph of the Section titled “ **The emergence of COVID-19 in late 2019**” with a plain word. Please double-check your punctuation, grammar, and syntax throughout the article.

 5. You should provide a brief explanation of force majeure, preferably following the first line of the introduction, and also show why COVID-19 serves as an exemplary case of force majeure.

 6. Finally, the article, also in Section 2, includes the following quote in the second paragraph:

 “Twenty countries, representing approximately 50% of the global population, account for over 80% of the estimated global excess mortality for the January 2020 to December 2021 period. These countries are Brazil, Colombia, Egypt, Germany, India, Indonesia, the Islamic Republic of Iran, Italy, Mexico, Nigeria, Pakistan, Peru, the Philippines, Poland, the Russian Federation, South Africa, the United Kingdom of Great Britain and Northern Ireland, Turkey, Ukraine, and the United States of America (USA).”

 I do not believe that Nigeria's inclusion among the countries representing 80% of global excess mortality due to COVID-19 is accurate. Firsthand experience suggests that Nigeria was one of the African nations that experienced significantly fewer deaths from the virus.

Is the case presented with sufficient detail to be useful for teaching or other practitioners?

Yes

Is the work clearly and accurately presented and does it cite the current literature?

Yes

If applicable, is the statistical analysis and its interpretation appropriate?

Not applicable

Are all the source data underlying the results available to ensure full reproducibility?

Yes

Are the conclusions drawn adequately supported by the results?

Yes

Is the background of the case’s history and progression described in sufficient detail?

Yes

Reviewer Expertise:

Law generally, and more specifically- force majeure, energy law and policy (oil, gas, renewables, natural resources, critical minerals, global energy transitions, energy justice, sustainable energy, and ocean energy), voluntary and regulated carbon markets, carbon capture, utilisation and storage (CCUS), climate change law and policy (mitigation, adaptation, climate justice, climate litigation, corporate climate governance, and international climate finance), international law (public and private), international environmental law and policy (a focus on key international agreements), human rights, international trade, and ADR, focusing on international arbitration.

I confirm that I have read this submission and believe that I have an appropriate level of expertise to confirm that it is of an acceptable scientific standard, however I have significant reservations, as outlined above.

# Reviewer report: Reviewer response for version 1

**DOI:** 10.21956/openreseurope.20763.r52271

**Published:** 2025-04-04

**Authors:** Michael Jopling

**Affiliations:** 1 University of Brighton, Brighton, England, UK

This case study report outlines the challenges of undertaking a citizen science project, aimed at training citizen scientists to participate in a research project examining online tracking and data privacy, during the COVID-19 pandemic and how they were overcome. The report is impressively honest about the researchers' self-professed naivety in not having expected the move online to affect the project as much as it did, given its online focus. The report outlines the methods used and the levels of participation achieved and concludes with convincing implications for large-scale citizen science activities, especially during such force majeure events.  

 Inevitably, the report begs a number of questions, such as how representative were the anonymised comments collected in Box 1? How did project leaders explain the relatively low rates of both MOOC completion and "conversion" of participants into becoming citizen scientists? And how far can the results can be applied to citizen science activities more widely? In fact, more consideration of the limitations of the research would have been welcome, but this should not detract from an interesting and important project examining key aspects of data privacy.

Is the case presented with sufficient detail to be useful for teaching or other practitioners?

Yes

Is the work clearly and accurately presented and does it cite the current literature?

Yes

If applicable, is the statistical analysis and its interpretation appropriate?

Not applicable

Are all the source data underlying the results available to ensure full reproducibility?

Yes

Are the conclusions drawn adequately supported by the results?

Yes

Is the background of the case’s history and progression described in sufficient detail?

Yes

Reviewer Expertise:

Digital learning and teaching, aspects of citizen (social) science

I confirm that I have read this submission and believe that I have an appropriate level of expertise to confirm that it is of an acceptable scientific standard.

# Reviewer report: Reviewer response for version 1

**DOI:** 10.21956/openreseurope.20763.r52081

**Published:** 2025-03-21

**Authors:** Giorgio Di Pietro

**Affiliations:** 1 European Commission- Joint Research Centre, Seville, Spain

Thank you for the opportunity  to comment this work. It nicely summarises the activities and results of a project aimed at providing citizens with the necessary knowledge and skills required to assess GDPR compliance. This is indeed a crucial topic in today’s world.

 I have a few suggestions in an attempt to clarify and add some information.

 First, I would like to encourage the authors to briefly describe the initially planned activities for the project that had to be cancelled due to the advent of the pandemic. Was it possible to find a good replacement for all the cancelled activities (including the most important ones)?

 Second, I have several concerns about participation in the ‘massive open online course’ (MOOC) developed by the project. The number of participants is not very large.  How can one explain this? Are there any mechanisms that have been put in place to ensure participation among the more vulnerable segment of the population? Has the outbreak of Covid-19 influenced the demographic composition of the participants?

 Third, to evaluate the effectiveness of the MOOC developed by the project, it would be important to test whether participants were able to correctly apply the knowledge and skills learned in this course. Can/has this be done?  

 Fourth, it would be interesting to know whether the results of the investigations (over 1350) made by citizen scientists have led to any tangible results (e.g., companies correcting errors in GDPR compliance or companies paying more attention in complying with GDPR).

 Overall, it is a nice research, but more information about the actual impact of the project could be presented.

Is the case presented with sufficient detail to be useful for teaching or other practitioners?

Partly

Is the work clearly and accurately presented and does it cite the current literature?

Yes

If applicable, is the statistical analysis and its interpretation appropriate?

Not applicable

Are all the source data underlying the results available to ensure full reproducibility?

Partly

Are the conclusions drawn adequately supported by the results?

Yes

Is the background of the case’s history and progression described in sufficient detail?

Partly

Reviewer Expertise:

My area of expertise is education

I confirm that I have read this submission and believe that I have an appropriate level of expertise to confirm that it is of an acceptable scientific standard, however I have significant reservations, as outlined above.
